# Debut century in cardiac surgery - Is it worth mentioning

**DOI:** 10.1186/1749-8090-10-S1-A300

**Published:** 2015-12-16

**Authors:** Kalyana Javangula

**Affiliations:** 1 Leeds Teaching Hospitals NHS Trust, Leeds, LS1 3EX, UK

## Background/Introduction

Consultant surgical practice including the outcomes has been under great scrutiny from day 1. Having a solid foundation in the early stages is going to give confidence to take high risk cases in the later years with good results. With this I am going to present my own series of first 100 cases.

## Aims/Objectives

Here I would like to present, how I performed first 100 operations as a consultant surgeon without mortality, stroke, re-exploration, deep sternal wound infections or mediastinits. I take this opportunity to share my methodology of case selection and preparation for surgery with the upcoming consultant colleagues and senior trainees who are going to be the future generation of cardiac surgeons.

## Method

This is prospective, which was retrospectively collected on my patients whom I operated between July 2010 and March 2011 as a new consultant. The total 105 cases includes 76 CABG, 21AVR, 8 AVR+CABG and third of these were acute cases. The mean age was 59.3 (5 - 84) and mean additive Euro score of 4.5 (0-10) and only 10% are mod -poor LV function. None of the patients had IABP either before or after surgery.

## Results

There were no post-op mortality or stroke and none of the patients were re-explored for bleeding or tamponade. Only 2 patients were readmitted to ICU for tachyarrythmias and 3 patients needed re-intubations (out of which one needed tracheostomy). 10% had minor postop complications including small left sided pleural effusions, pneumothorax and superficial leg wound infections. None of these patients had deep sternal or leg wound infections requiring vac therapy or rewiring.

## Discussion/Conclusion

Careful patient selection and attention to detail are the key factors for achieving good postoperative outcomes. Career as a consultant cardiac surgeon is a challenging journey. We are practising in times where outcomes are highly scrutinised and available to public domain. Gone those days where people are going to accept mishaps (just because you started as a newly appointed consultant). Good foundation in the initial stages gives you the confidence to handle challenging and high risk cases in the future.

